# Advancing clinical MRI exams with artificial intelligence: Japan’s contributions and future prospects

**DOI:** 10.1007/s11604-024-01689-y

**Published:** 2024-11-16

**Authors:** Shohei Fujita, Yasutaka Fushimi, Rintaro Ito, Yusuke Matsui, Fuminari Tatsugami, Tomoyuki Fujioka, Daiju Ueda, Noriyuki Fujima, Kenji Hirata, Takahiro Tsuboyama, Taiki Nozaki, Masahiro Yanagawa, Koji Kamagata, Mariko Kawamura, Akira Yamada, Takeshi Nakaura, Shinji Naganawa

**Affiliations:** 1 Department of Radiology, The University of Tokyo, 7-3-1 Hongo, Bunkyo, Tokyo Japan; 2 Department of Diagnostic Imaging and Nuclear Medicine, Kyoto University Graduate School of Medicine, Sakyoku, Kyoto Japan; 3 Department of Radiology, Nagoya University Graduate School of Medicine, Nagoya, Aichi Japan; 4 Department of Radiology, Faculty of Medicine, Dentistry and Pharmaceutical Sciences, Okayama University, Kita-Ku, Okayama, Japan; 5 Department of Diagnostic Radiology, Hiroshima University, Minami-Ku, Hiroshima City, Hiroshima Japan; 6 Department of Diagnostic Radiology, Tokyo Medical and Dental University, Tokyo, Japan; 7 Department of Artificial Intelligence, Graduate School of Medicine, Osaka Metropolitan University, Abeno-Ku, Osaka, Japan; 8 Department of Diagnostic and Interventional Radiology, Hokkaido University Hospital, Sapporo, Hokkaido Japan; 9 Department of Nuclear Medicine, Hokkaido University Hospital, Sapporo, Hokkaido Japan; 10 Department of Radiology, Kobe University Graduate School of Medicine, Chuo-Ku, Kobe, Japan; 11 Department of Radiology, Keio University School of Medicine, Tokyo, Japan; 12 Department of Radiology, Osaka University Graduate School of Medicine, Suita, Osaka Japan; 13 Department of Radiology, Juntendo University Graduate School of Medicine, Tokyo, Japan; 14 Medical Data Science Course, Shinshu University School of Medicine, Matsumoto, Nagano Japan; 15 Department of Diagnostic Radiology, Kumamoto University Graduate School of Medicine, Kumamoto, Kumamoto Japan

**Keywords:** Artificial intelligence, Magnetic resonance imaging, Healthcare, Medicine, Review

## Abstract

In this narrative review, we review the applications of artificial intelligence (AI) into clinical magnetic resonance imaging (MRI) exams, with a particular focus on Japan’s contributions to this field. In the first part of the review, we introduce the various applications of AI in optimizing different aspects of the MRI process, including scan protocols, patient preparation, image acquisition, image reconstruction, and postprocessing techniques. Additionally, we examine AI’s growing influence in clinical decision-making, particularly in areas such as segmentation, radiation therapy planning, and reporting assistance. By emphasizing studies conducted in Japan, we highlight the nation’s contributions to the advancement of AI in MRI. In the latter part of the review, we highlight the characteristics that make Japan a unique environment for the development and implementation of AI in MRI examinations. Japan’s healthcare landscape is distinguished by several key factors that collectively create a fertile ground for AI research and development. Notably, Japan boasts one of the highest densities of MRI scanners per capita globally, ensuring widespread access to the exam. Japan’s national health insurance system plays a pivotal role by providing MRI scans to all citizens irrespective of socioeconomic status, which facilitates the collection of inclusive and unbiased imaging data across a diverse population. Japan’s extensive health screening programs, coupled with collaborative research initiatives like the Japan Medical Imaging Database (J-MID), enable the aggregation and sharing of large, high-quality datasets. With its technological expertise and healthcare infrastructure, Japan is well-positioned to make meaningful contributions to the MRI–AI domain. The collaborative efforts of researchers, clinicians, and technology experts, including those in Japan, will continue to advance the future of AI in clinical MRI, potentially leading to improvements in patient care and healthcare efficiency.

## Introduction

The integration of artificial intelligence (AI) in magnetic resonance imaging (MRI) has led to significant improvements in clinical MRI exams [1, 2]. MRI is highly valued for its absence of ionizing radiation and its excellent soft tissue contrast, making it indispensable for diagnosing a wide range of diseases across various organs and systems [3]. However, challenges such as long acquisition times, motion sensitivity, patient comfort, and technical complexities persist. AI has shown promise in addressing these issues by optimizing each step of the MRI process, ranging from patient preparation and scan planning to image reconstruction and assistance in image interpretation and report generation.

Japan’s unique characteristics in MRI examinations, including a large number of MRI scanners per capita [4, 5], skilled technologists [6], extensive health screening data [7], combined with the national health insurance system [8] provides a fertile ground for AI research and development. Japan boasts one of the highest densities of MRI scanners globally, enabling widespread access to advanced imaging technology. Additionally, Japanese MRI technologists are renowned for their expertise [6], contributing to high-quality image data collection. This, coupled with the country’s national insurance system that offers CT and MRI scans to all citizens [7] ensures the collection of unbiased imaging data across all age groups and socioeconomic statuses. Collaborative research across multiple universities is also prevalent in Japan, with initiatives like Japan Medical Imaging Database (J-MID) enabling the sharing of large datasets [9], further fostering AI development. These factors collectively create an ideal environment for developing and implementing AI solutions in clinical MRI. Furthermore, Japan is actively contributing to the education of AI applications across various organs and modalities [10–17], showcasing its commitment to this field.

**Fig. 1:**
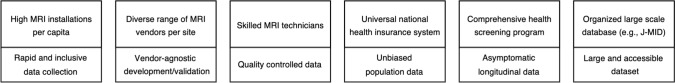
Japan’s unique characteristics in clinical MRI examinations

In this narrative review, we provide the applications of AI into MRI with a focus on Japan’s contribution to this field. The article first discusses the role of AI in improving clinical MRI exams, covering each step from optimizing scan protocols and patient preparation to improving image quality and postprocessing. Additionally, AI’s role in clinical decision-making, particularly in segmentation and radiation therapy planning, as well as the use of large language models (LLMs) for reporting will be discussed. Given the extensive research on AI in MRI, this paper predominantly focuses on studies from Japan. In the latter half, the paper offers insights into Japan’s potential future contributions to the field (Fig. 1), mentioning initiatives such as the J-MID and identifying possible areas for research and development that could advance the current state of technology.

## Patient preparation and scan planning

MRI examinations often necessitate the acquisition of multiple sequences, leading to prolonged scan times [18]. Ensuring optimized protocols for every patient can be challenging, especially in institutions where radiologists may not always be readily available. AI algorithms are proving valuable in optimizing scan planning by automatically determining ideal slice positioning and coverage, leading to more efficient scan sessions [19–21]. These approaches not only ensure greater consistency across different operators but also tailors the acquisition process to each patient’s unique anatomy.

Additionally, active research is exploring the optimization of MRI sequences themselves using AI, going beyond adjustments to higher order parameters like matrix size and echo times. Examples include utilizing deep reinforcement learning for designing radiofrequency pulse [22] and generating k-space sampling patterns [23]. While these technical advancements are beyond the scope of this review, they represent an exciting frontier in AI-driven MRI development.

## Image reconstruction

Deep learning reconstruction (DLR), particularly those utilizing convolutional neural networks (CNNs), have demonstrated success in MRI reconstruction [24, 25]. These AI algorithms can effectively increase image resolution, reduce noise, and subsequently allow for more undersampled acquisitions. Numerous studies have shown its promise in clinical MRI exams, including but not limited to the brain [26–28], head and neck [29], upper abdomen [30–33], prostate [34], musculoskeletal [35], and breast. These studies, along with many others reported daily worldwide, lead to a broad agreement that DLR effectively improves subjective image quality and signal-to-noise ratio in structural imaging compared to other existing acceleration techniques.

Beyond these improvements in image quality, it is essential to evaluate the broader impact of DLR on patient management. A critical aspect to consider is its influence on clinical assessments, such as T-category evaluation [36], which directly affects patient care decisions. In a cohort of 213 patients pathologically diagnosed with non-small cell lung cancer, the application of DLR improved image quality but did not necessarily lead to changes in T-category evaluation [37]. Similarly, in prostate imaging, while DLR enhanced image quality, it did not result in alterations to Prostate Imaging Reporting and Data System classification [38]. These findings underscore the importance of evaluating the clinical implications of DLR, rather than focusing solely on improvements in image quality that may not directly translate into clinical value.

Quantitative measurements, such as apparent diffusion coefficient (ADC) and relaxation time, derived from images reconstructed with DLR are an area that warrants further exploration, despite the widespread reporting of its effectiveness in structural imaging. For example, one study found no substantial differences in prostate ADCs derived with and without DLR [39]. In contrast, another study reported significantly higher ADC values in both prostate tissue and lesions when comparing images reconstructed with DL to those with parallel imaging [40]. Additionally, the effectiveness of DLR has been demonstrated in non-structural imaging, such as diffusion metrics [41], relaxation [42, 43], and neuromelanin imaging [44], with findings suggesting that DLR enables more aggressive undersampling. Given the diversity of DLR techniques and implementations, their impact on quantitative measurements remains a topic of significant interest.

While the majority of the techniques discussed above rely on image-to-image CNNs with supervised learning, exciting developments are actively emerging, including physics-informed DLR approaches such as unrolled variational networks [45] and self-supervised learning methods [46]. Physics-informed DLR approaches leverage k-space information and provide higher acceleration and theoretically less chance of hallucination. Notably, some unrolled variational networks have been validated in patient cohorts, demonstrating promising results even at high undersampling rates, such as *R* = 12 [40, 47–49]. Additionally, self-supervised learning methods are gaining traction as powerful tools for image reconstruction [43, 46]. These techniques have demonstrated great technical potential, and further clinical validations in clinical settings are underway.

## Image postprocessing: segmentation and contrast synthesis

### Segmentation

Image segmentation has been one of the most promising areas of AI in medical imaging. Deep learning models, particularly U-Net and its variants, have dramatically improved the speed and accuracy of MRI segmentation tasks. These AI-driven segmentation tools can automatically delineate organs, tumors, and other structures of interest, saving significant time for radiologists and improving consistency [50]. As segmentation precision and accuracy increase, the segmented volumes and volumes of interest can be subsequently used for downstream tasks such as classification and prognosis (e.g., longitudinal volume tracking for evaluating treatment response) [51].

One of the most successful applications of MRI segmentation is brain segmentation, due to the brain’s complex anatomy and functional importance. Brain segmentation software, such as FreeSurfer, has been widely used in neuroimaging and brain analyses [52]. These pipelines involve computationally intensive, time-consuming optimization steps. Deep learning-based neuroimaging pipelines for the automated segmentation of structural human brain MRI scans, including surface reconstruction and cortical parcellation, have been proposed [53, 54].

Segmentations with AI are not only useful for diagnosis but also potentially improve and optimize radiation treatment planning [55]. AI-driven segmentation and image analysis tools are streamlining the radiation therapy planning process by accurately delineating target volumes and organs at risk in MRI images. These algorithms can automatically contour tumor volumes and critical structures with high accuracy, reducing the time-consuming manual delineation process. For example, an AI-based automatic pipelines enabled precise lesion segmentation and subsequent outcome prediction using pretreatment MRI [56, 57].

### Contrast synthesis

Contrast synthesis represents an exciting application of AI in MRI. Generating missing contrast-weighted images from existing sequences can allow for the retrieval of important contrast information, even after the exam has concluded, thereby improving workflow flexibility and potentially improving patient outcomes. For example, not all institutions have MR angiography (MRA) routinely acquired in their protocol, and using AI to retrospectively generate MRA [58] or increase the spatial resolution of MRA [59] may be helpful in certain cases. Similarly, synthesizing fat-suppressed images in the spine [60] and in the breast [61, 62] has been demonstrated. Further, Liu et al. have demonstrated that synthesizing a wide range of contrast weighted images from available contrast weighted images is feasible [63].

Reducing the intravenous dosage of contrast agents are also being explored, by boosting the subtle T1 shortening effects of the gadolinium based contrast agent. This may enable to reduce contrast dosage while preserving imaging quality. For example, AI-enhanced MRI exams with 10% dosage showed similar to improved image quality and contrast enhancement compared to standard dosage [64, 65].

The combination of MRI and AI offers the capability to synthesize not only a diverse range of MRI contrasts but also images from other modalities. PET imaging, for instance, offers powerful insights into metabolic processes, but it carries drawbacks such as high cost and invasiveness of radioactive tracer administration, and limited availability. MRI, which is more accessible and non-invasive, can be combined with AI to infer specific PET images. For example, a study has shown that AI-based models can generate synthetic methionine PET images that strongly correlate with real PET images, demonstrating good performance for glioma grading and prognostication [66]. Applications to amyloid-beta PET imaging have also been explored. For example, Vega et al. demonstrated that amyloid-beta PET images can be synthesized from structural MRI with a high degree of similarity to the real PET images [67]. Additionally, Fujita et al. utilized MR fingerprinting [69] to synthesize amyloid-beta PET images that showed significant correlation with Aβ-PET measurements and cognitive scores [68].

## Clinical decision making: detection and classification

### Detection

Detecting lesions on medical images is the first and most crucial step in diagnosing a wide range of medical conditions. Without the initial identification of these lesions, healthcare providers would be unable to diagnose or manage patient care effectively. For instance, the detection of an incidental, unruptured intracranial aneurysm on an MRI can guide patients towards appropriate monitoring or even preventive intervention.

One of the earliest reports on the development and validation of AI for detecting cerebral aneurysms in time-of-flight MRA in a clinical setting came from Japan. In a study by Ueda et al., a retrospective evaluation included 748 aneurysms for training, 649 for validation, and 80 for an external test dataset, demonstrating high sensitivity and improved aneurysm detection compared to initial reports [70]. Similarly, Nakao et al. demonstrated that combining a convolutional neural network with a maximum intensity projection algorithm is effective for detecting intracranial aneurysms [71]. This combination of convolutional neural network and maximum intensity projection has also been applied in dynamic contrast-enhanced (DCE) breast imaging. Adachi et al. demonstrated that their AI system showed higher performance in detecting lesions in maximum intensity projections of DCE breast MRI and improved the diagnostic performance of human readers [72].

While the use of AI system has demonstrated promising performance in medical literature, several key aspects of its integration into the interpretation of MRI exams, such as the timing of its involvement (e.g., concurrent with the initial review, prescreening, second reader, or stand-alone), and its potential impact on factors like interpretation time and the short- and long-term interaction between AI and human readers, are yet to be fully evaluated. A systematic investigation of these factors is essential to optimize the integration of AI into clinical practice, ensuring accurate and efficient image interpretation while maintaining patient safety and trust in the technology.

### Classification

AI can potentially assist clinicians in providing metrics for classification of the lesion, patient survival, predicting treatment response [73, 74], or assessing the risk of disease recurrence. One of the earliest reports on using CNNs to classify diffuse liver disease from MRI was reported from Japan: a retrospective study with 634 patients with various liver fibrosis evaluated with gadoxetic acid-enhanced hepatobiliary phase magnetic resonance (MR) imaging demonstrated that a deep convolutional neural network model has high diagnostic performance in the staging of liver fibrosis [75]. Although this study did not include an external dataset, it became a pioneering work in using CNNs to classify lesions from MRI such as for breast lesion classification from DCE [76, 77], and also for discrimination of soft tissue tumor [78].

From a clinical workflow perspective, classification and stratification are considered more downstream tasks compared to lesion detection. These tasks are closer to the decision-making and management phases of patient care, where the outcomes directly influence treatment plans and patient prognosis. Because the results of classification and stratification have a more immediate impact on patient outcomes, integrating AI systems into these stages of clinical practice requires greater caution and thorough validation.

### Assisting in reporting and communication

Large Language Models (LLMs) are transforming radiology reporting by assisting and generating reports [79]. AI-powered reporting systems, based on LLMs, can automatically generate preliminary radiology reports from MRI findings. These systems can ensure consistent terminology, include all relevant measurements and comparisons, and even flag critical findings for immediate attention. Chat Generative Pre-trained Transformer (ChatGPT) achieved a diagnostic accuracy rate of 50% in neuroradiology cases [80] and 94% in brain tumor cases [81]. However, these studies were based on text inputs (i.e., the image findings were described by humans as inputs) and the performance is expected to be dependent on how the image findings were described by human readers. A recent study has further evaluated multimodal LLMs with image itself as inputs [82]. Limitations of LLMs that currently limit applications in radiology include hallucinations, knowledge cutoff dates, poor complex reasoning, and a tendency to perpetuate bias [79]. The impact of LLMs are impacting radiology dramatically and for further information we recommend review articles solely on LLMs and radiology as in [79].

## Japan’s contributions, core strengths, and future role

### Japan’s contribution in MRI research

Japan has made significant contributions to MRI research, leveraging its technological prowess and unique healthcare landscape. The Japanese Society for Magnetic Resonance in Medicine (JSMRM) is a non-profit society established in 1986, with more than 3600 members in the fields of clinical medicine, basic medicine, physics, chemistry, biology, and engineering [83]. JSMRM is one of the oldest and largest national societies in the field of magnetic resonance in medicine worldwide.

Notable recent contributions of JSMRM in clinical MRI include topics such as diffusion MRI [84, 85], quantitative MRI [51, 86], and the glymphatic system [87–89]. It is noteworthy that many key studies on the glymphatic system have been reported from Japan, such as the in vivo observation of gadolinium-based contrast agent leakage and its mechanism of deposition and accumulation in the brain [87]. A practical tool for obtaining a surrogate metric to evaluate the glymphatic system, called diffusion tensor image analysis along the perivascular space (DTI-ALPS), has also been proposed from Japan [88]. This technique has shown associations between MRI parameters and amyloid β deposition, neuronal changes, and cognitive impairment in Alzheimer's disease [89].

The JSMRM includes members from industry, reflecting the country's strong tradition in electronics and imaging technology. Combined with its advanced healthcare system, this has fostered strong academic-industry collaborations [90–98].

### Japan’s core strengths and its potential impact

Japan's distinct landscape in MRI examinations creates a uniquely advantageous environment for the development and implementation of AI technologies (Fig. 1). With the highest per capita number of MRI installations worldwide [4, 5], sourced from various vendors, Japan has the capacity to rapidly accumulate large MRI datasets. Additionally, Japan’s national health insurance system is one of the few globally that provides universal access to CT and MRI scans [8], allowing for the collection of imaging data across all demographics, irrespective of age or socioeconomic status. This inclusivity is crucial in minimizing bias during AI development for MRI [99]. Japanese MRI technologists, known for their exceptional proficiency, consistently produce high-quality images, leading to the generation of extensive, quality controlled MRI data sets. This convergence of widespread MRI accessibility, diverse patient representation, and technical expertise forms an ideal foundation for advancing AI applications in MRI.

These extensive MRI data are systematically collected and organized into well-structured medical imaging datasets. A significant initiative in this area is the J-MID project [9]. Established in 2018 with the backing of the Japan Agency for Medical Research and Development, J-MID functions as a cloud-based national database infrastructure dedicated to diagnostic imaging. It provides researchers with access to a substantial repository of high-quality imaging data, which is critical for developing and validating robust AI algorithms in medical imaging. Currently, this comprehensive database contains over 500 million images, including CT and MRI scans, as well as diagnostic reports from both national and private university hospitals across Japan.

In addition to scans covered by national insurance, Japan offers a distinctive health screening program [7]. This elective program allows individuals to undergo additional tests, including MRI, beyond the standard scans provided by insurance. Notably, many participants in the program undergo multiple annual screenings, resulting in the generation of valuable longitudinal data [100]. Unlike many other research databases, this health screening program database includes a substantial number of healthy individuals [101], including young adults. This unique aspect allows researchers to explore the early stages of disease development and assess the potential for preventive interventions. The predominance of healthy participants also reduces the confounding effects of pre-existing conditions, making it easier to isolate specific factors influencing health outcomes. The combination of longitudinal data and the unique characteristics of the program participants makes this database an invaluable resource for advancing medical knowledge and enhancing healthcare practices. These elements together create an optimal environment for developing and implementing AI solutions in clinical MRI.

### Future research and development directions

Japan’s aging population presents unique opportunities for the application of AI in MRI, especially when combined with the country’s core strengths. While increasing the number of physicians could address healthcare shortages [102], Japan is exploring alternative solutions that leverage its technological expertise and data integration capabilities without expanding the physician workforce.

Centralizing available AI-based tools will enhance their accessibility by allowing users to easily identify and utilize existing options. To support this goal, the Japanese Radiological Society has established guidelines for medical image AI, requiring vendors to be certified and registered in their database. In Japan, hospitals are reimbursed for using AI-powered diagnostic support software, provided they implement appropriate safety management based on guidelines established by relevant academic societies [103, 104]. Additionally, creating guidelines for AI integration will ensure its seamless incorporation into clinical practice.

Developing standardized, scalable AI applications necessitates datasets from various types of scanners. The presence of multi-vendor equipment within Japanese facilities facilitates the evaluation of AI’s generalizability across different platforms [105]. Japan is uniquely positioned to foster collaboration across diverse platforms, enabling the development of AI solutions that are compatible with a wide range of systems.

Furthermore, Japan’s universal healthcare system enables the rapid collection of high-quality data across the entire population, including extensive multimodal datasets from healthy individuals. These datasets are crucial for training AI models that are both accurate and unbiased. By capitalizing on these strengths, Japan can address the high medical demand and physician shortages, positioning itself at the forefront of global MRI-AI innovation.

## Conclusion

Japan, with its technological expertise and unique healthcare system, is well positioned to be a global leader in the MRI–AI domain. The collaborative efforts of researchers, clinicians, and technology experts worldwide, with significant contributions from Japan, will shape the future of AI in clinical MRI, ultimately leading to improved patient outcomes and more efficient healthcare systems.
